# Supplementary material for: VEGFA, B, C: Implications of the C-Terminal Sequence Variations for the Interaction with Neuropilins
Source: Biomolecules. 2022 Feb 26;12(3):372. doi: 10.3390/biom12030372 (PMC8945599; doi:10.3390/biom12030372)
Supplement: Supplementary file 1 [file biomolecules-12-00372-s001.zip › biomolecules-1543635-supplementary.pdf]

Supplementary Figure S1: Thermal shift assays

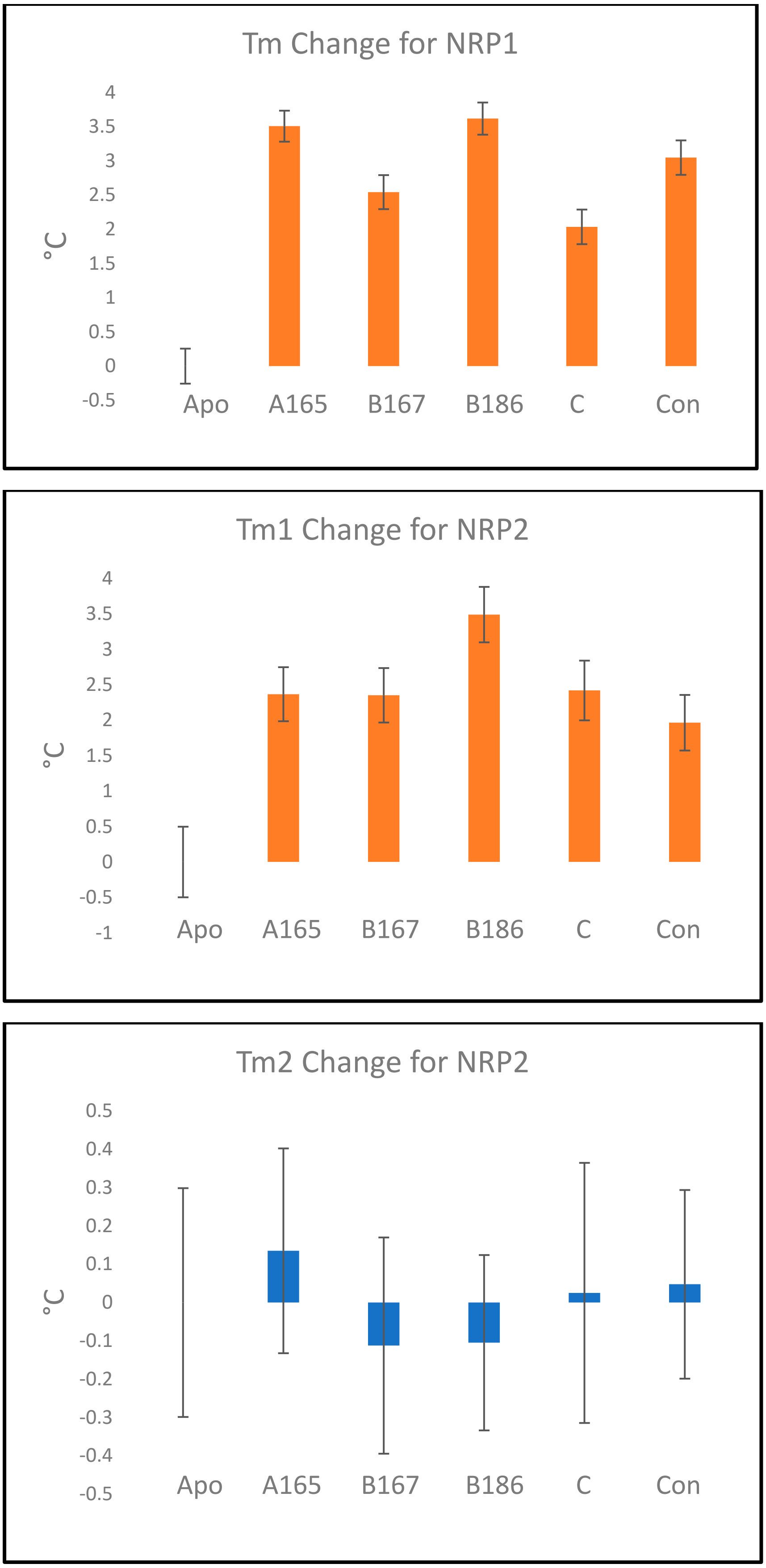

Supplementary Figure S2A:

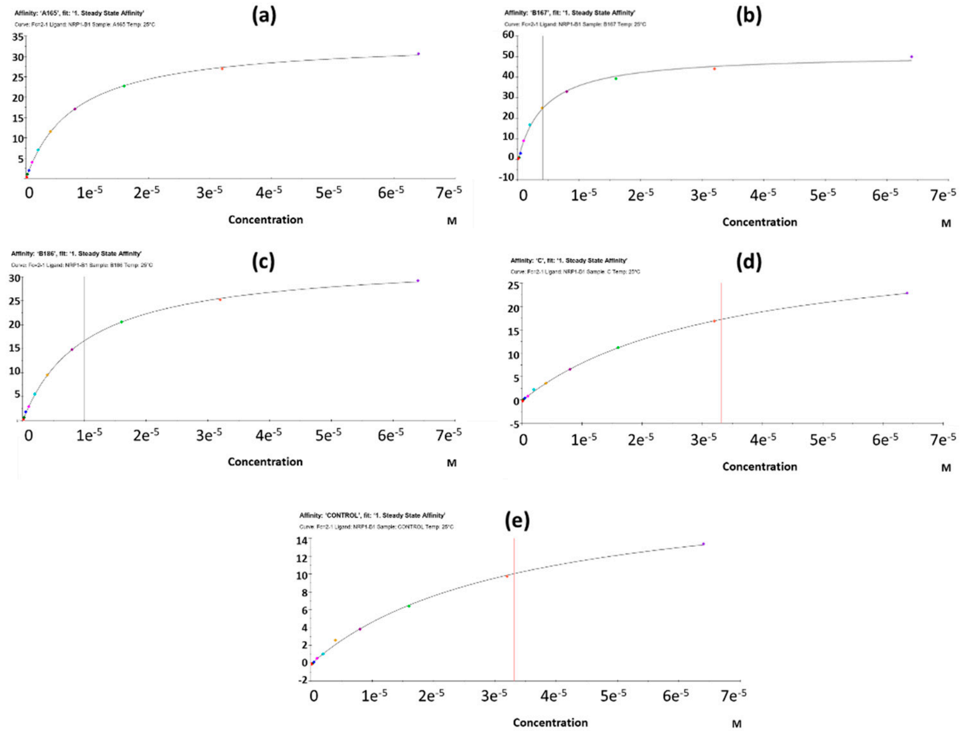

SPR data for NRP1b1 with peptides (a) A165, (b) B167, (c) B186, (d) C and (e) Con.

Supplementary Figure S2B:

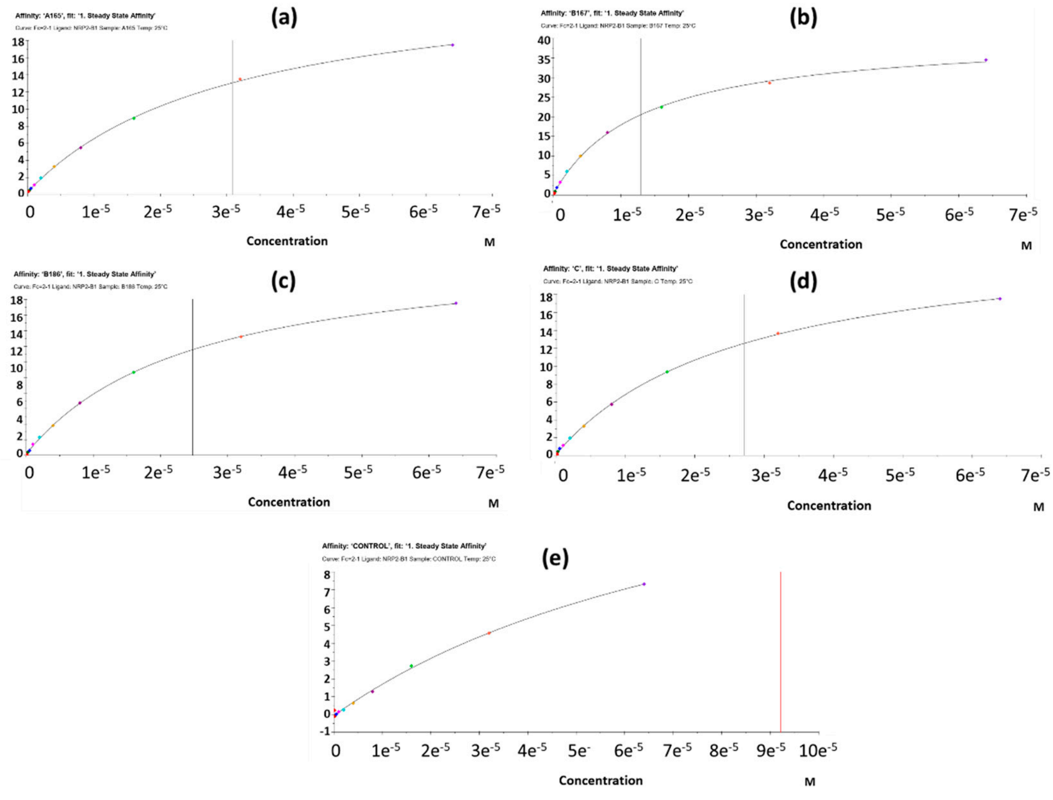

SPR data for NRP2b1 with peptides (a) A165, (b) B167, (c) B186, (d) C and (e) Con.

Supplementary Figure S3

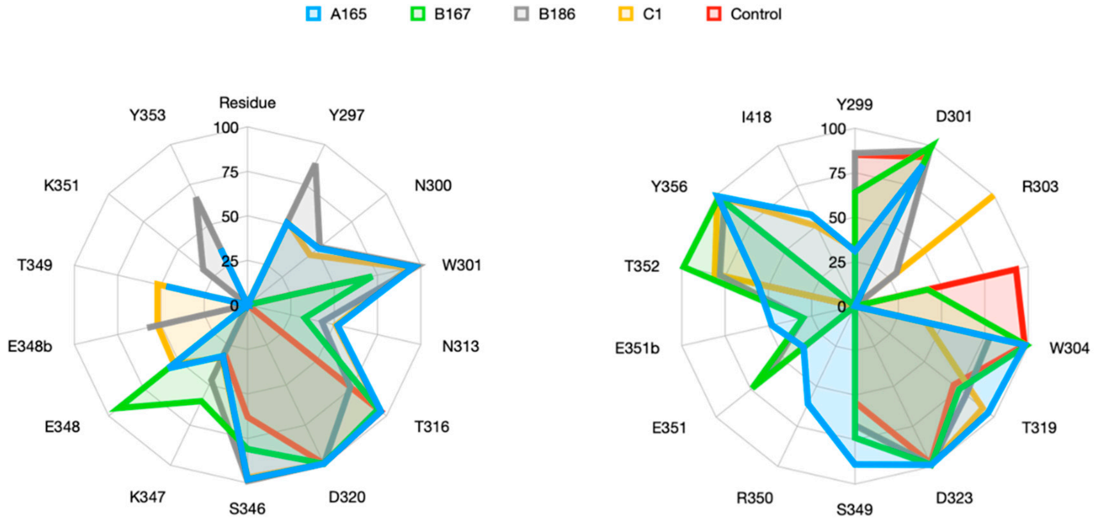

Radar plot of table 4

Radar plot of table 5

Radar plots created based on the frequencies included in Table 4 and Table 5.

Supplementary Figure S4

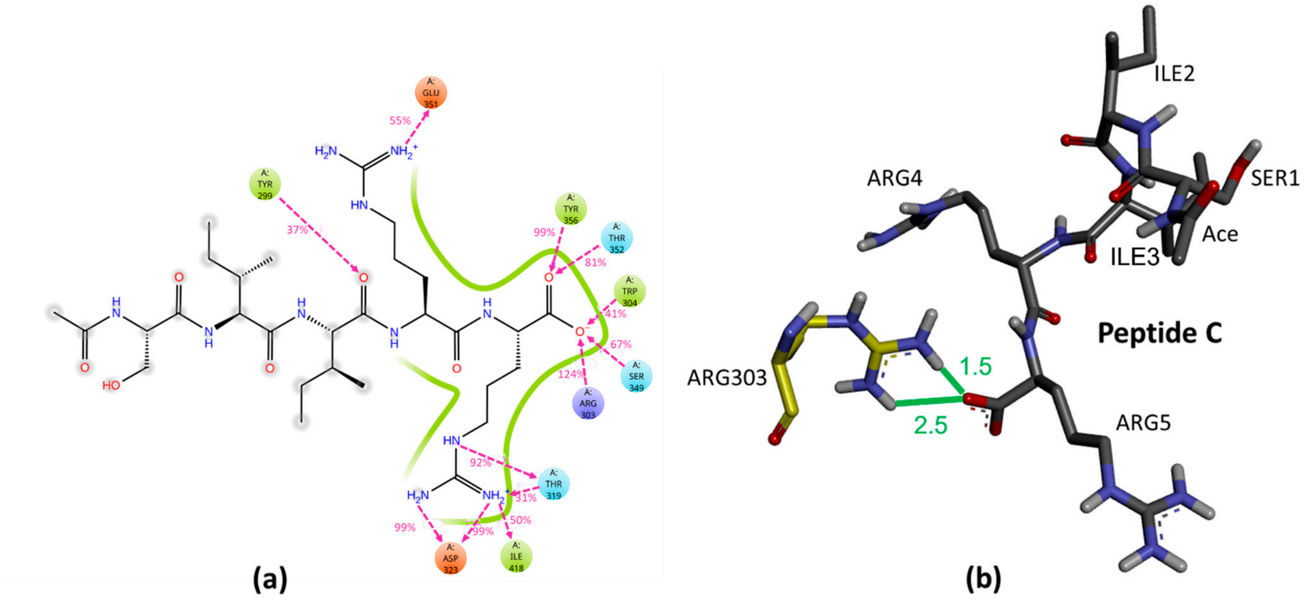

Interactions between NRP2-b1 Arg303 and the peptide C, as identified in MD simulations. (a) The ligand-centric summary of all the contacts identified between peptide C and NRP2 depicted in the form of a 2-D ligplot diagram. Interaction strength is quantified by the frequency of occurrences in the trajectory filtering out rarely occurring events and focussing on more important interactions.

In the plot, the light green circles depict hydrophobic residues and interactions, light blue is associated with the polar side chains while charged residues are denoted by blue and red colour for the positive and negative side-chains, respectively. Interactions are indicated by dashed arrows. In case of the Arg303, one or more hydrogen atoms of its guanidinium group can form hydrogen bonds with the C-terminal carboxylic group, indicating that the interactions between peptide C and Arg303 can be observed for the duration of the simulation. (b) Stick model of the peptide C showing interaction with the Arg303 that was captured during the MD simulations.
